# A proof-of-concept framework for the preference elicitation and evaluation of health informatics technologies: the online PRESENT patient experience dashboard as a case example

**DOI:** 10.1186/s12911-020-1098-z

**Published:** 2020-05-24

**Authors:** Emmanouil Mentzakis, Daria Tkacz, Carol Rivas

**Affiliations:** 1 Economics Department, School of Social Sciences, University of Southampton, Southampton, UK; 2 Faculty of Health Sciences, University of Southampton, Southampton, UK; 3 Social Science Research Unit, Department of Social Science, University College London (UCL), 18 Woburn Square, London, WC1H 0NR UK

**Keywords:** Discrete choice experiment, DCE, Online dashboard, Technology development, Health informatics, Healthcare, Cost-benefit analysis, Willingness-to-pay, Ranking, Checklist

## Abstract

**Background:**

Constrained budgets within healthcare systems and the need to efficiently allocate resources often necessitate the valuation of healthcare interventions and services. However, when a technological product is developed for which no market exists it is a challenge to understand how to place the product and which specifications are the most sought after and important for end users. This was the case for a dashboard we developed, displaying analyses of patient experience survey free-text comments.

**Method:**

We describe a customisation and evaluation process for our online dashboard that addresses this challenge, using a Discrete Choice Experiment (DCE). We were not interested in the exact content of the dashboard, which was determined in previous stages of our larger study, but on the availability of features and customization options and how they affect individuals’ purchasing behaviours.

**Results:**

Our DCE completion rate was 33/152 (22%). Certain features were highly desirable - the search function, filtering, and upload own data - and would contribute significant added value to the dashboard. Purchasing behaviour was dependent on the dashboard features, going from a 10 to 90% probability to purchase when we moved from a baseline to a fully-featured dashboard.

The purchasing behaviour elicited in this study assumes individuals already have buy-in to the online dashboard, so we assessed only how the various features of our dashboard influence the probability of purchasing the product. Results were used to inform development of a generic checklist of desirable healthcare dashboard features as well as to refine the dashboard itself. Our study suggests the development of the online dashboard and its roll-out in the market would result in a positive net benefit in terms of utilities. The cost-benefit analysis offers a lower bound estimate of the net benefit as it does not acknowledge or incorporate non-monetary benefits that would result from the use of the online dashboard, such as from improved healthcare management.

**Conclusion:**

DCEs can be successfully used to inform development of an online dashboard by determining preferences for particular features and customisation options and how this affects individuals’ purchasing behaviours. The process should be transferable to the development of other technologies.

## Background

Constrained budgets within healthcare systems and the need to efficiently allocate resources often necessitate the valuation of healthcare interventions and services. For economists, markets provide the main mechanism through which the true value of a good is revealed. When someone chooses to purchase a good/service, this implies a preference, whereby the utility (or satisfaction) the person derives from their purchase is at least as big as the cost/price they had to pay. However, what happens when preferences need to be considered and understood for a good/service for which there is no current market? This is the problem we had when developing a new patient experience online dashboard. Our dashboard involved novel use of text analysis and text analytics of national health service patient experience survey free-text comments in a way that could drive healthcare improvements. For such non-market goods, two further dimensions need consideration. First, a market could eventually exist but often the developer requires some prior insight of the market structure so as to place their product correctly. Second, a product could have an impossibly large number of different specifications, and choosing which to supply, i.e. which features are the most sought after and important for eventual users, requires prior intelligence during the development stage. Such information is not just important for market placement, but also in order to develop a system that best satisfies its users, which was a core ethos of the PRESENT study [1].

In the larger PRESENT study we had sought to determine these factors from a literature review, survey of relevant potential end users of the dashboard, a workshop and interviews. But the review findings were limited, due to a lack of previous work in the field of evaluations of healthcare dashboard feature preferences, the survey produced comments on what respondents had personal experience of, rather than what they desired, and the qualitative data from the workshops and interviews was a retrospective co-construction between researchers and participants [2].

We believed that Discrete Choice Experiments (DCEs) would provide a different way of getting at the information, that would satisfy all our requirements and that would allow elicitation of monetary values not only of the product but of each individual feature. DCEs are based on primary experimental data and provide researchers with the ability to place individuals in controlled environments (situations) and investigate issues in isolation without potential confounding biases. DCEs are founded on the consumer behaviour and preferences theory of Lancaster [3] and Rosen [4] and draw on subconscious cognitive processes. They postulate that utility is not derived from the consumption of a good per se, but from the characteristics this good possesses. Thus, the characteristics of a good determine its value, and differences in the characteristics result in different degrees of desirability for the individual. In essence, the researcher describes a good in terms of its features, with various combinations of features available as if they were multiple different hypothetical products. Potential users are then asked which product version they would purchase if they were given the chance. The underlying intuition is that the user will consider the features of the different products presented to them and will trade-off among them, as well as the specific price for each product. They will then choose to purchase (or rather state their intention to purchase) the one they deem the most attractive or desirable given the price. DCEs so far have seen applications in many fields, among others including marketing, environmental, economics and health [5–7], and have been recommended in the evaluation of health technologies [8, 9], but to our knowledge they have not been used to develop healthcare technologies.

This paper sets out to describe a customisation and evaluation process for an online/software dashboard that was developed as part of an online toolkit (the ‘PRESENT’ study toolkit). Specifically, the DCE provides feedback on market placement and calculates how much individuals are willing-to-pay for each of the features of the online dashboard and how likely they are to purchase or not purchase a dashboard given its features. This knowledge was useful for determining the final design of the PRESENT dashboard, particularly when it came to making choices between different conflicting information from the prior review, survey and qualitative work, which we summarise further later in this paper. The context and methodology are directly applicable to the development and evaluation of any physical or electronic product and service and this paper aims to also serve as the introduction of such an approach in the product development and evaluation of health care technology more generally. Our work has been welcomed by our networks as much needed.

## Prior work and context

UK healthcare policy foregrounds the patient’s perspective on quality of care. The PRESENT study was developed to display results from the successful National Cancer Patient Experience Survey (CPES). Since 2010 this has been sent over a 3-month period each year to all patients treated for cancer as in-patients or day cases in NHS Trusts in England. But there has been no way of systematically, efficiently and usefully analysing and reporting the free-text responses in this and other similar surveys, despite this being a recognised need. The PRESENT project therefore used rule-based information retrieval [10] to structure CPES free-text comments and Python programming language (https://www.python.org/) to display results and text analytics in a summary visual format in a digital dashboard display that could be drilled down to the original free-text.

A dashboard approach was chosen because of its popularity in healthcare. In 2009, the NHS Connecting for Health initiative, now incorporated within The Health and Social Care Information Centre (http://webarchive.nationalarchives.gov.uk/20130502102046/http://www.hscic.gov.uk/aboutus), began the Clinical Dashboards Programme. This was an attempt to make dashboard development more accessible to healthcare professionals as a useful way of summarising healthcare-relevant data. But its recommendations do not appear to have been based on domain-specific considerations that incorporated patient views and nor do they appear to have been informed by empirical work on healthcare dashboard usability and design considerations. Moreover this programme was so flexible that it led to very divergent design approaches that often breached Gestalt design principles [11]. Good dashboards should tailor their designs to specific end user requirements without breaking these principles. Dowding and colleagues [12], in a 2015 review of the use of clinical and quality dashboards in healthcare environments, found some indication of their effectiveness in improving patient outcomes. However these authors stated that further high quality detailed research studies were needed to confirm this and to establish guidelines for dashboard design for healthcare settings. They also noted heterogeneity in the design of the dashboards they evaluated, for example concerning the use of text or graphs, colours, and how information was presented to users.

The PRESENT study therefore aimed to develop a dashboard that could drive service improvements whilst maintaining Gestalt principles, and to provide guidelines on healthcare dashboard design. To do so, we used an interdisciplinary approach that maintained the patient voice in the data, whilst producing themes and displays that were meaningful to staff in terms of healthcare improvement possibilities, and concordant with programming and web design principles. The study was divided into three stages. The first stage (see Table 1 for findings) led to development of a list of potential attributes of a dashboard, which was refined in subsequent stages of the study and used for the DCE. This stage involved a scoping literature review on clinical digital toolkit design which found little relevant literature; what there was, focused on design principles rather than applied use. It also included a stakeholder dashboard scoping survey, disseminated through relevant healthcare networks and websites; *n* = 33 of the 35 respondents were healthcare professionals. From this survey we identified several attributes additional to those determined from the review (Table 1).

**Table 1:** Summary of the key features healthcare dashboards and toolkits should incorporate according to our prior literature review and our survey of healthcare professionals

Feature category	Examples
Access	minimising workload burden, e.g.: • easily digested summaries (s) • integrated into an already existing system / constantly in sight • registration absent or clear and short engaging homepage with clear statement of value real-time access e.g. during clinics access to the raw data (s)
Flexibility and individualisation	personalisation features alerts and reminders information pins dynamic movement of elements of the dashboard to fit around the user’s focus and workflow ensure reactive to different devices layout logic and route that anticipates the user’s workflow a combination on one computer screen of high-level overviews to highlight problem areas and benchmarks of regional and national performance of choice (s) the potential to upload and incorporate their own quantitative data (s)
Use of images and videos	informative rather than decorative images, that reflect the user’s demographic profile videos to instruct on how to use the dashboard
Chart types	line and bar charts – well labelled- for analysing relationships, tables for extracting specific values and complex tasks; function to choose graphic type or table including reference data points such as national averages for easier interpretation several graphs on one screen
Data interrogation	ability to filter the data in real-time and to sort it by any level and quality indicator; filter parameters need to be practical and clearly defined and aligned with the user’s work feedback that the page has changed after filtering search box for interrogating the data / drop-down list or dictionary of suggested search terms
Print and export	the option to print information, download data outputs
Community features	patient stories and other narrative videos forum, chat room or similar community feature signposting to other sources of information and support
Governance	security and privacy prioritised good data reliability (s)
Offering recommendations and solutions	highlighting problems, but also offering recommendations or solutions a simple predictive tool (s)

Items marked (s) were identified from our survey of healthcare professionals. All other items were identified from our review

In the second study stage our focus was on developing several prototype dashboards based on results from stage one. The prototypes were discussed with 34 stakeholders in patient experience (patients, healthcare staff and third sector) in our six workshops (*n* = 4–9 per group). Modally, these were female, in their 50s, and they self-reported as from a cross-section of ethnic groups; Table 2. Different types of stakeholder had different understandings of dashboard usage. In the first workshops we proposed that we would produce more than one final dashboard, to represent different user settings, but it rapidly became clear that potential end-users wanted one system with different layers of access depending on who they were. This is in keeping with CPES; the comments in this survey cut across primary and secondary care of all types. The focal feature was the patient experience and different end uses could be satisfied simply by including filters and search functions within one dashboard. The prototypes were further explored in semi-structured interviews with purposively selected workshop participants (those across the three main stakeholder groups who diverged from the main group or had strong viewpoints). Interview theme saturation was reached at *n* = 12 participants.

**Table 2:** Breakdown of pre-DCE workshop discussants by characteristics

**Stakeholder role (*n*= 34)**	**Number (%)**
Patient (predominantly cancers and in one group multiple sclerosis, but also heart disease; diabetes mellitus, heart and retinopathy; non-cancer muscular or skeletal conditions and dementia; sickle cell disease; mental health; sarcoma)	14 (41)
Carer	1 (3)
Consultant/specialist	1 (3)
GP	1 (3)
Nurse	3 (9)
Quality team member	0 (0)
Variety of commissioning roles	2 (6)
Budget holder	0 (0)
Non-executive director NHS patient and public engagement lead	1 (3)
Policy-maker	0 (0)
Academic	2 (6)
Other (an advocate for dementia patients; patients who were also carers/budget holders/academics)	7 (20)
Did not say	2 (6)
**Gender**	
Male	8 (23)
Female	23 (68)
Did not say	3 (9)
**Ethnicity**	
White British	19 (58)
Any other white background	3 (9)
Mixed/multiple ethnic groups: white and black Caribbean/African	2 (6)
Asian/Asian British: Indian	1 (3)
Asian/Asian British: Chinese	1 (3)
Any other Asian background	2 (6)
Black: African	1 (3)
Black: Caribbean	1 (3)
Did not say	3 (9)
**Ages**	**Year of birth**
Minimum	1938
Maximum	1988
Mean	1965
Mode	1984

**Fig. 1:**
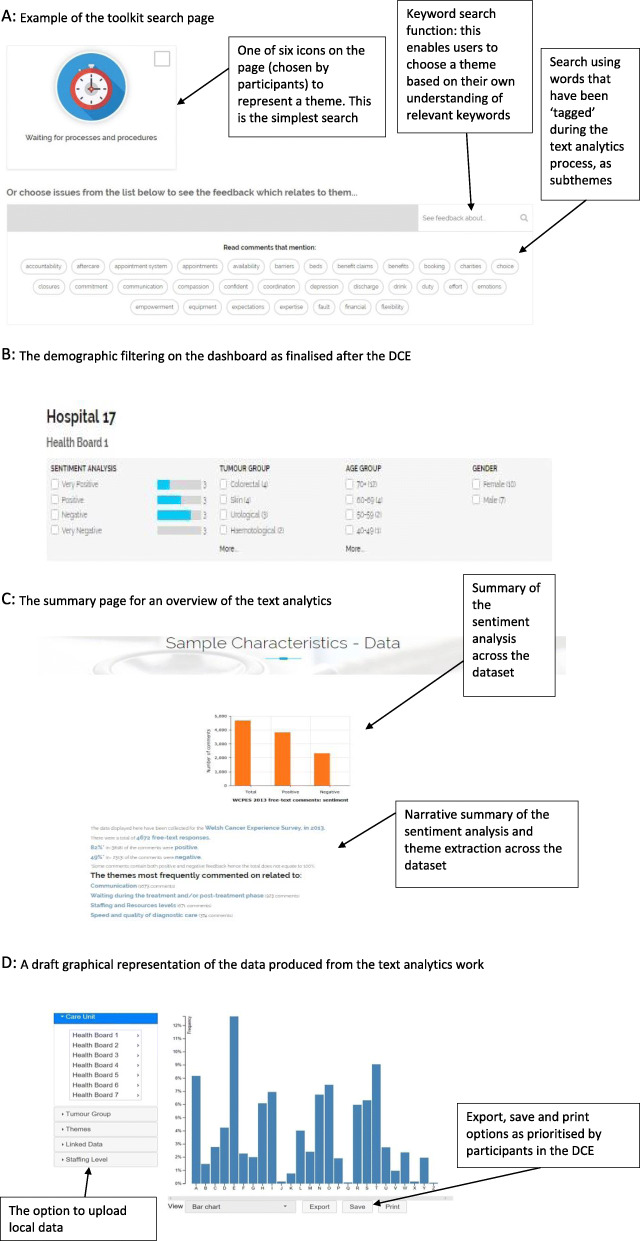
Details from our toolkit in development. **a** Example of the toolkit search page. **b** The demographic filtering on the dashboard as finalised after the DCE. **c** The summary page for an overview of the text analytics. Narrative summary of the sentiment analysis and theme extraction across the dataset. **d** A draft graphical representation of the data produced from the text analytics work. The option to upload local data. Export, save and print options as prioritised by participants in the DCE

From the workshops and interviews we determined data sharing issues, expectations around machine learning accuracy and patient experience survey sampling biases as critical topics for further debate and consideration before patient feedback data can be fully and optimally used. The workshops and interviews led to amendments to our prototype, such as level of granularity and filtering (see Fig. 1) as well as the decision to have one prototype suitable for all, rather than multiple versions.

The DCE was a part of our final proof-of-concept and validation stage of the main study and was followed by amendments to our prototype and then walkthrough techniques, to explore usability and implementation of the dashboard.

The research questions for the DCE were:

1. How much are individuals willing-to-pay for each of the features of the online dashboard?
2. Which features are monetarily the most desirable, as a proxy for practical desirability in use by health services managers within cancer care?
3. How likely are they to purchase or not purchase a dashboard given its features?

## Methods

In conducting our DCE a number of steps needed attention so as to maximize the quality of responses and results and to provide answers to our research questions. These steps are now considered.

### Determining attributes and levels for our DCE

In this section we describe how we determined the attributes and levels for our DCE.

i. First, the features (called attributes) of the good in question need to be selected, that will be included in the DCE. Commonly these attributes are based on expert knowledge and discussion, literature reviews and often intuition. In the PRESENT study they were developed from our stage one and two work and a key reason for deciding on the DCE was to answer research question 2 (see above) and triangulate responses with those from other parts of the study.
ii. A price attribute may also be included in the DCE for willingness-to-pay (WTP) calculations, in other words, to determine the price one is willing to pay for a specific feature (research question 1).
iii. Subsequently, for each attribute, the number of levels of detail and the levels themselves have to be chosen. They should be realistic and cover the range of potential values within which an individual's preferences would fall. For example, for the attribute ‘search functionality’, three levels might be: no search; search by one fixed variable; search by selecting from a menu of suggested variables.

Our stage one and two work identified 21 potential attributes that were relevant to the prototype dashboard design that we had developed. Through discussion within the research team, and further co-design work – exploring the dashboard and attributes list in a workshop with our advisory group and through their networks within the DCE work - the number of attributes was reduced down to 10 key features of a healthcare dashboard.

### Experiment design for our DCE

**Fig. 2:**
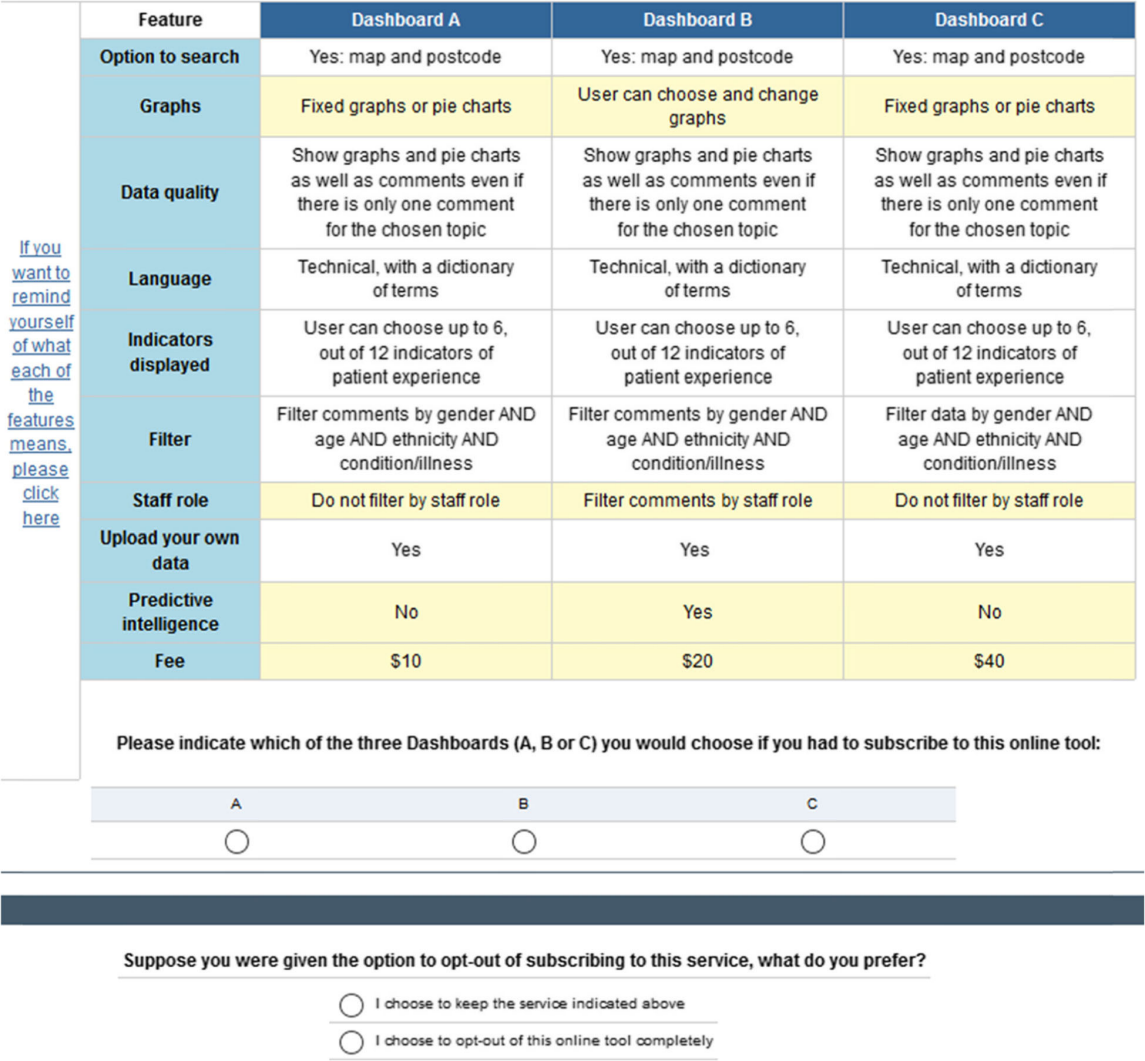
Example of choice set presented to individuals. Screenshot generated from iSurvey, the University of Southampton’s survey generation and research tool

By combining one level of each attribute for all attributes, we created a complete specification of the product with alternatives. In a DCE these alternatives are then placed in groups of two or three to create the scenarios (or choice sets) that are presented to respondents and that form the basis of the questionnaire. The number of alternatives and choice sets depends on the number of attributes and levels [5, 13, 14]. The procedures to determine which attribute levels to combine into alternatives and which alternatives to be placed together in choice sets is guided by experimental design principles that ensure efficiency and identification of intended effects (for more information on this see [13, 14]). For the online dashboard we had 8 attributes of 2 levels and 2 attributes of 4 levels, which required each respondent to make choices for a minimum of 15 choice sets of two alternatives each (i.e. 15 pairwise sets of two alternative hypothetical dashboards). Figure 2 presents an example of a choice set that was used in the survey.

Draft DCE pages were piloted online, using a questionnaire design, on three healthcare professionals who fitted the profile of potential users (health service managers and decision-makers), to help with the design, wording and presentation of the survey. We also elicited from potential users the maximum they would be willing to pay for a desired dashboard, and whether this should be costed as an individual or team purchase. Changes were implemented, such as avoiding repetitive wording and simplifying the comparisons, and further feedback sought via cognitive interviews (in the questionnaire development use of the term) with two stakeholders who were academic clinicians with decision-making responsibilities [1]. These ‘think aloud’ interviews led us to simplify the introductory pages and add screenshots of the dashboard that users could check at any time as a reminder. The number providing feedback was less than we had hoped for (our aim was 15 overall), due to project time constraints. However various studies have shown that 5–15 participants of any demographic make-up who are able to use technology are sufficient to find most potential issues with a technology and we achieved the bottom end of this range [15]. The final list of attributes and levels selected for the DCE is provided as Supplementary information. The cognitive burden of such choices was deemed large in feedback and a number of steps were taken to reduce it and render the DCE more manageable for respondents. First, we increased the number of alternative dashboards within each choice set to three. This reduced the number of choice sets required per respondent to eight. Second, a partial profile design was implemented, where only a subset of four attributes was allowed to vary among the three alternatives in each choice set but with a different subset of attributes across different choice sets. This convenience and discount in cognitive burden comes however with the cost that each respondent must see a larger number of choice sets. Using one of the features of our experimental design software, three different versions of this basic design were created that varied the combination of attributes and levels presented to individuals. This increased the number of choice sets that were evaluated by respondents to increase coverage of the full factorial, more accurately identify trade-offs and improve the overall efficiency of the experimental design. As such, three D-efficient fractional factorial partial-profile designs with eight choice sets each and three alternatives per choice set were generated. Each respondent was randomly assigned to one of the three versions.

Forcing individuals to indicate their preferred alternative out of the three in each choice ensures that individuals trade off between the attributes of the alternatives without the possibility of choosing to decline the purchase. However, this makes it impossible for the researcher to elicit true purchasing behaviour given that the corresponding real-word choice would be first whether to purchase a dashboard service and subsequently which version one would like. As such, we implemented a two-stage elicitation strategy where individuals were first asked which of the three alternatives they prefer and subsequently whether they would rather keep their preferred alternative or opt-out if given the chance to. This addresses research question 3.

All experimental designs were constructed using JMP software [16].

### Survey administration

The experiment was platformed on the iSurvey toolkit available from the University of Southampton. Over 200 stakeholders were invited to participate through our existing professional networks and Macmillan Cancer Care and their digital team. We contacted all of the clinical commissioning groups in England as well as a number of cancer charities. In addition, though a smaller and overlapping pool of potential respondents, the professionals who participated in other stages of the study, had previously shown interest in the study, or had attended our launch event, were invited and encouraged to share the link with colleagues. The questionnaire was also disseminated via members of our advisory group and steering committee, and shared within the Faculty of Health Sciences at the University of Southampton. The questionnaire was open for nine weeks, with one reminder. Ethics approval was obtained from an NHS National Research Ethics Committee (NRES 15/NS/0104) and the study was sponsored by the University of Southampton.

### DCE analysis

Analysis of DCE data assumes random utility theory and a linear-in-parameters utility function. When independent and identically distributed extreme value type I distributed error terms are assumed McFadden’s conditional logit (CL) arises [17]. This models the probability that an individual will choose a specific alternative from a set of three alternatives. Given that individual respondent characteristics do not change within a choice set, they cannot directly influence such probability. The coefficients (e.g. *β*_*k*_ for attribute-level *k*) estimated in such models denote the increase/decrease in utility obtained from an alternative possessing the characteristic *k*.

Relaxing some conditional logit assumptions, choices are further modelled through a nested logit (NL) model where alternatives are first grouped into nests (i.e. purchase or not-purchase) and the probability that an individual chooses an alternative from a set of available alternatives within a specific nest is modelled [18]. Again individual respondent characteristics cannot affect the choice of the alternative but can affect the choice of the nest (i.e. purchase or not-purchase).

#### Willingness-to-pay values

Following estimation of models and coefficients for each of the attribute levels, willingness-to-pay values can be calculated. As discussed previously, coefficients depict part-worth utility and as such present the relative importance of attribute levels. By taking ratios of coefficient one can calculate the marginal rates of substitution (MRS) between them, i.e. how much of an attribute level an individual is willing to lose to gain more of another attribute level. Incorporating a price component in the attributes allows one to translate such ratios into their monetary representations, i.e. WTP values. WTPs represent the amount of money an average individual is willing to pay to obtain a service/product that has a specific feature attribute level.

#### Predicted probabilities

Further inference on the results can be drawn from calculating predicted probabilities. These probabilities represent the likelihood that an individual will purchase a given product or not, or the change in the likelihood of purchasing a product or not when its features change. Specifically, we present three types of predicted probabilities.

- The first one is used to provide better insight into the magnitude of the effect of each attribute level (i.e. how much it contributes to the attractiveness of the product). This may be termed dashboard desirability. For this we define a baseline product whose features are given in Table 3. Subsequently we create alternative dashboards that are identical to the baseline with the exception of only one attribute level. For instance, one alternative dashboard might be identical to the baseline except that it possesses the additional capability for the user to upload their own data into the dashboard (i.e. attribute: **Upload own data** – Yes). The possible configurations (i.e. the number of possible alternative products) are large so, without any loss of generality, we restrict ourselves to changing only one attribute level at a time, which results in a total of 12 alternative dashboards. The probability of purchase of each of these alternative dashboards is then calculated and compared to the baseline. This difference indicates the change in the predicted probability of purchase when one attribute level changes with respect to the baseline. Given that we have intentionally chosen the baseline dashboard to contain the least desired level of each feature, with the exception of price, where it is the cheapest, probability differences are expected to be positive, implying that the more desired features there are, the greater the probability of selecting the dashboard.
- The second type of predicted probabilities follows a similar logic but gives an assessment of the importance of each attribute level in preventing an average individual from opting-out of purchasing the dashboard tool. In this case, we calculate the probability of opting-out for the baseline as well as all other alternative dashboards and we again compare them. More desirable features are expected to reduce the probability of opting-out. Given that in these calculations the presence of the opt-out as an option is necessary, this is only possible for the second stage of the DCE choice. Recall that we implement a two-stage elicitation strategy where individuals are first asked which of three alternative dashboard they prefer and subsequently they are offered the option to keep their preferred alternative or opt-out altogether.
- The third type calculates the unconditional predicted probability of purchase and of opt-out (i.e. declining purchase) for three hypothetical dashboards, i.e. the baseline, a fully-featured low price and a fully-featured high price alternative. Such figures give a sense of the overall tendency to purchase in the sample of respondents and can aid in building market scenarios that can guide market placement and revenue streams.

**Table 3:** Characteristics of the baseline alternative in the DCE

Attribute	Baseline value
**Search**	No, Only regional data visible
**Graphs**	Fixed graphs or pie charts
**Data resolution**	Show graphs and pie charts as well as comments even if there is only 1 comment for the chosen topic
**Language**	Technical, with a dictionary of terms
**Indicators displayed**	Fixed indicators of patient experience shown at the same time
**Filter**	Filter only by condition or illness (e.g. type of cancer)
**Staff role**	Do not filter by staff role
**Upload own data**	No
**Predictive intelligence**	No predictive intelligence capability
**Annual subscription fee**	£250

## Results

Of the 152 individuals invited, 148 logged into the survey (i.e. a possible response rate of 97%, though we are unable to determine a true response rate as we asked them to pass the link on). Of those, 116 responded to at least one choice set but did not finish the survey so that their data could not be used. In total, 32 respondents completed the DCE, giving a completion/retention rate of about 22%. While identification of coefficients does not rest on sample size (i.e. the experimental design ensures that enough information is available to estimate parameters), the generalisability or robustness of the findings would need to be verified, given this retention rate.

### Descriptive statistics

Before we look at the estimation results we discuss the make-up of the sample (Table 4). Overall, the mean age was 49 years with 81% females and 38% working for the NHS. Enquiring about information on individual circumstances related to NHS posts, band 7 was most common (accounting for 50% of respondents), while management (58% of respondents) was the most common professional area. Individuals with academic roles comprised 53% of respondents; again this means findings need to be further verified with healthcare staff though most academics were also part-time clinicians or on local commissioning boards.

**Table 4:** Descriptive statistics of the collected sample (*n* = 32)

**Age**	Mean	Min	Max
	48.5	30.0	77.0
	Frequency	Percentage	
**Sex**			
Male	6.0	18.8	
Female	26.0	81.3	
**NHS staff**			
No	20.0	62.5	
Yes	12.0	37.5	
**Band/grade**			
Band 7	6.0	50.0	
Band 8a	2.0	16.7	
Band 8b	1.0	8.3	
Band 8d	2.0	16.7	
Other	1.0	8.3	
**Professional area**			
Clinical psychology	1.0	8.3	
Management	7.0	58.3	
Medicine/surgery	1.0	8.3	
Nursing	1.0	8.3	
Other	1.0	8.3	
Physiotherapy/Occupational therapy	1.0	8.3	
**Area of specialty**			
Commissioning	1.0	9.1	
Communications and Engagement	1.0	9.1	
Complaints, Patient experience and Risk Manager	1.0	9.1	
Diabetes and Obesity	1.0	9.1	
Innovation evaluation and implementation	1.0	9.1	
Musculoskeletal	1.0	9.1	
Obstetrics and Gynaecology	1.0	9.1	
Operational / Commissioning	1.0	9.1	
Service Development Manager	1.0	9.1	
Urology	1.0	9.1	
Communications and patient experience	1.0	9.1	
**Role**			
Academic	9.0	52.9	
Charity	1.0	5.9	
Health Researcher	1.0	5.9	
Healthwatch	1.0	5.9	
Professional	1.0	5.9	
Community engagement	1.0	5.9	
Local authority officer	1.0	5.9	
Manager of charity	1.0	5.9	
Patient	1.0	5.9	

### Estimation results

Table 5 presents the estimation result from three models. The conditional logit can be used to estimate the first stage of the DCE question (i.e. forced-choice without an opt-out) and the first and second stage of the DCE questions combined (i.e. three alternative dashboards plus an opt-out to choose from). Combinations of first and second stage are also analysed using a nested logit. Results for all three are given in Odds Ratios which indicate how likely one is to choose an alternative that features an attribute level compared to the baseline (research question 2).

**Table 5:** Estimation results of the DCE. Data generated using JMP software Version 13.0

	Conditional Logit – Forced choice	Conditional Logit – Opt-out	Nested Logit
**(a) Attribute estimates**			
**Search** Yes, choose a hospital from a drop down list	3.792*** (1.796)	2.763*** (1.040)	6.321*** (4.241)
**Search** Yes, keyword search	2.962** (1.386)	2.539*** (0.915)	4.730** (2.939)
**Search** Yes, map and postcode	2.948** (1.423)	2.318*** (0.742)	3.007 (2.059)
**Graphs** User can choose and change graphs	1.627* (0.407)	1.666** (0.361)	1.848** (0.508)
**Data resolution** Show graphs and pie charts only if more than 6 responses, but show all comments	1.232 (0.302)	1.331 (0.299)	1.317 (0.434)
**Language** Lay, that is no jargon	1.895** (0.561)	1.125 (0.232)	1.714 (0.569)
**Indicators display** User can choose up to 6, out of 12 indicators of patient experience	1.424* (0.305)	1.221 (0.226)	1.576 (0.437)
**Filter** Filter data by gender AND age AND ethnicity AND condition/illness	3.703*** (0.907)	3.193*** (0.699)	5.023*** (1.647)
**Staff role** Filter comments by staff role	1.798** (0.454)	1.568*** (0.247)	2.033** (0.618)
**Upload own data** Yes	2.596*** (0.723)	1.742*** (0.355)	2.823*** (1.119)
**Predictive intelligence** Predictive intelligence capability to inform and help plan capacity	1.144 (0.261)	1.144 (0.275)	1.144 (0.356)
**Fee**	0.999*** (0.0002)	0.999*** (0.0002)	0.999*** (0.0002)
Decline the service (Opt-out)		8.823*** (4.574)	
**(b) Probability to decline the service**			
Age			0.994 (0.029)
Sex (1 if female)			29.35*** (29.29)
NHS staff			1.322 (0.891)
Constant			0.0506 (0.092)
# of respondents	33		

Robust standard errors in parentheses; *** *p* < 0.01, ** *p* < 0.05, * *p* < 0.1

Common patterns appear across models. Being able to ‘Filter data’ (by gender AND age AND ethnicity AND condition/illness) seems to be the most influential feature, followed by the ability to ‘Search’ (a hospital from a drop-down list). All three search features are highly desirable for respondents, while almost equally important is the ability to ‘Upload one's own data’. Further, ‘Filtering comments (by staff roles)’ and ‘Graphs customisation’ also appear important, while ‘Use of lay language’ and ‘Indicators display’ only achieve limited significance. Interestingly, ‘Data resolution’ and ‘Predictive intelligence’ were not identified as important in any of the models even though identified as desirable in our scoping survey. Finally, cost of the dashboard was highly significant, suggesting that respondents were sensitive to price changes as predicted by economic theory. Overall, on average, individuals were very likely to decline to purchase the baseline service (i.e. almost 9 times more likely to decline purchase than to purchase), while females were much more likely to do so as indicated by the nested logit model (research question 3).

### WTP monetary valuations

Table 6 presents WTP calculations (research question 1). Given that WTP are essentially non-linear combinations of the estimated coefficients, statistical significance and relative size closely follows the coefficient results. All three models point to an identical list of important attribute levels albeit there is some variation in the magnitude of the elicited WTP. This is largely explained by the different model assumptions and other model features, and in the vast majority, WTPs are not statistically different across the three models.

**Table 6:** Willingness-to-pay values from the DCE estimations. Data generated using JMP software Version 13.0

	Conditional Logit – Forced choice	Conditional Logit – Opt-out	Nested Logit
**Search** Yes, choose a hospital from a drop down list	1673.75 (2.19)**	1836.33 (2.34)**	2446.80 (2.37)**
**Search** Yes, keyword search	1363.52 (2.06)**	1683.55 (2.33)**	2062.10 (2.42)**
**Search** Yes, map and postcode	1357.49 (2.01)**	1518.86 (2.41)**	1460.96 (1.67)*
**Graphs** User can choose and change graphs	611.41 (1.57)	922.53 (1.57)	815.25 (1.61)
**Data resolution** Show graphs and pie charts only if more than 6 responses, but show all comments	261.75 (0.78)	516.10 (1.11)	365.61 (0.77)
**Language** Lay, that is no jargon	802.76 (1.89)	212.45 (0.61)	715.41 (1.56)
**Indicators display** User can choose up to 6, out of 12 indicators of patient experience	443.44 (1.63)	360.70 (1.24)	603.35 (1.76)*
**Filter** Filter data by gender AND age AND ethnicity AND condition/illness	1643.74 (2.85)***	2097.37 (2.65)***	2141.81 (2.55)**
**Staff role** Filter comments by staff role	736.55 (2.13)**	812.63 (2.06)**	941.73 (2.03)**
**Upload own data** Yes	1197.61 (2.29)**	1002.80 (2.01)**	1376.96 (2.04)**
**Predictive intelligence** Predictive intelligence capability to inform and help plan capacity	168.78 (0.54)	243.31 (0.51)	178.27 (0.40)

z-statistics in parentheses; *** *p* < 0.01, ** *p* < 0.05, * *p* < 0.1

Overall, respondents are willing-to-pay £1674 to £2447 (conditional and nested logit) for their most preferred ‘Search’ feature (i.e. drop down list of hospitals), followed by ‘Filter (by age, gender and condition)’ which is valued at £1644 to £2142. The other two ‘Search’ options, by keyword and map/postcode, are valued at £1364 to £2062 and £1357 to £1461, respectively. ‘Capability of uploading own data’ was valued at £1198 to £1377 and finally, ‘Filter by staff role’ was valued at £736 to £941.

We should note that although the rest of the attribute levels do not appear as significant, this does not imply they are not desired in the dashboard. As discussed previously, coefficients convey a relative piece of information, namely how much more a feature is desired compared to the baseline or to the baseline level. As such, statistically insignificant WTP values imply that respondents did not systematically value dashboards with these attribute levels more than they valued dashboards with the respective bassline attribute level. For instance in the case of ‘Graphs’ (with two levels: a) ‘Fixed graphs or pie charts’ and b) ‘User can choose and change graphs’) an insignificant WTP suggests respondents were not willing to pay more for a dashboard with the user specified graphs compared to a dashboard with fixed graphs. This is vastly different from the notion that graphics are altogether not important to the dashboard users.

### Predicted probabilities calculations

The results from the predicted probabilities calculations are given in Table 7. Recall that the values in the table are changes in predicted probabilities and as such interpreted as a percentage point change. Positive values imply an increase in the chances of purchasing an alternative dashboard level (i.e. identical to the baseline dashboard in all aspects apart from the specific attribute indicated) compared to the baseline dashboard. Correspondingly, negative values suggest the attribute level evaluated is less desired than the respective baseline attribute level and as such the chances of purchasing the alternative dashboard are lower than those of the baseline dashboard (research question 3).

**Table 7:** Predicted probabilities calculations from DCE estimations. Data generated using JMP software Version 13.0

	Conditional Logit – Forced choice	Conditional Logit – Opt-out		Nested Logit	
	Difference in predicted probability from Baseline	Difference in predicted probability from Baseline	Change in predicted probability of opt-out	Difference in predicted probability from Baseline	Change in predicted probability of opt-out
**Search** Yes, choose a hospital from a drop down list	32.2	10.9	−9.1	27.9	−4.2
**Search** Yes, keyword search	26.4	9.7	−8.1	23.8	−3.4
**Search** Yes, map and postcode	26.3	8.4	−7.0	16.8	−2.1
**Graphs** User can choose and change graphs	11.6	4.5	−3.7	9.0	−1.0
**Data resolution** Show graphs and pie charts only if more than 6 responses, but show all comments	4.8	2.3	−1.9	3.9	−0.4
**Language** Lay, that is no jargon	15.4	0.9	−0.7	7.8	−0.9
**Indicators display** User can choose up to 6, out of 12 indicators of patient experience	8.3	1.5	−1.3	6.5	−0.7
**Filter** Filter data by gender AND age AND ethnicity AND condition/illness	31.6	13.2	−11.0	24.7	−3.5
**Staff role** Filter comments by staff role	14.0	3.8	−3.2	10.5	−1.2
**Upload own data** Yes	23.2	4.9	−4.1	15.8	−2.0
**Predictive intelligence** Predictive intelligence capability to inform and help plan capacity	3.1	1.0	−0.8	1.8	−0.2
**Fee** £1500	−17.7	−3.6	3.1	−10.0	1.0

The Baseline is an alternative that has the following features:

**Search** - No, Only regional data visible

**Graphs** - Fixed graphs or pie charts

**Data resolution** - Show graphs and pie charts as well as comments even if there is only 1 comment for the chosen topic

**Language** - Technical, with a dictionary of terms

**Indicators displayed** - Fixed indicators of patient experience shown at the same time

**Filter** - Filter only by condition or illness (e.g. type of cancer)

**Staff role** - Do not filter by staff role

**Upload own data** - No

**Predictive intelligence** - No predictive intelligence capability

**Fee -** £250

All three models point to similar ranking across attribute levels, with the ‘Search’ feature of drop-down hospital list increasing the chances of an alternative being selected by 32 percentage points (p.p.) in the forced-choice and 28 p.p. in the NL. Equally large changes in likelihood are obtained by the ‘Filter (by age, gender and condition)’ feature, increasing chances by 32 p.p. and 25 p.p., respectively. The remaining ‘Search’ levels and ‘Upload own data’ capabilities follow. Looking at the price effect, an increase of the annual fee from £250 to £1500 drops the probability of purchase by about 18 p.p. in the forced choice and 10 p.p. in the NL.

Moving on to the second type of predicted probabilities where we calculate the change in the predicted probability of opting-out, negative values imply an attribute level will result in a lower probability to opt-out (i.e. they are a desired feature), whereas positive values suggest an increased chance of opting-out and not purchasing (i.e. an unattractive feature). For both the CL and NL models we see that the changes in the opt-out probabilities are relatively small, with about 13 p.p. and 3.5 p.p. drop in the CL and NL, respectively for ‘Filtering (by age, gender and condition)’. ‘Search’ features follow, with values between 7 p.p. and 9 p.p. for the CL and 4.2 p.p. and 2 p.p. for the NL, suggesting that all features are desirable and reduce the chances of opt-out (research questions 1, 2 and 3).

We note that such drops in the opt-out probability potentially appear small. However, this is to be expected given that there is a large tendency of respondents to opt-out of purchasing the baseline dashboard, which in essence is the least desirable dashboard that can be configured given the attributes specified. To gain insight into the overall attractiveness of the dashboard tool and the potential market demand, the third type of predicted probabilities is presented in Table 8. Individuals in the vast majority of cases (i.e. 91%) decline purchase of the baseline dashboard but will choose to purchase when a fully featured alternative is offered with 89% for the low and 80.5% for the high pricing options. From this we can also see the effect of price on a highly desirable dashboard, where an increase of £1250 (i.e. from £250 to £1500) reduces the probability of purchase by about 8.5 percentage points (research question 3).

**Table 8:** Predicted probabilities of purchase and opt-out for three representative dashboards. Data generated using JMP software Version 13.0

	**Baseline Dashboard**	**Fully-featured Low price**	**Fully-featured High price**
	**Search** No, Only regional data visible	**Search** Yes, choose a hospital from a drop down list	**Search** Yes, choose a hospital from a drop down list
	Fixed graphs or pie charts	User can choose and change graphs	User can choose and change graphs
	**Data resolution** Show graphs and pie charts as well as comments even if there is only 1 comment for the chosen topic	**Data resolution** Show graphs and pie charts only if more than 6 responses, but show all comments	**Data resolution** Show graphs and pie charts only if more than 6 responses, but show all comments
	**Language** Technical, with a dictionary of terms	**Language** Lay, that is no jargon	**Language** Lay, that is no jargon
	**Indicators displayed** Fixed indicators of patient experience shown at the same time	**Indicators display** User can choose up to 6, out of 12 indicators of patient experience	**Indicators display** User can choose up to 6, out of 12 indicators of patient experience
	**Filter** Filter only by condition or illness (e.g. type of cancer)	**Filter** Filter data by gender AND age AND ethnicity AND condition/illness	**Filter** Filter data by gender AND age AND ethnicity AND condition/illness
	**Staff role** Do not filter by staff role	**Staff role** Filter comments by staff role	**Staff role** Filter comments by staff role
	**Upload own data** No	**Upload own data** Yes	**Upload own data** Yes
	**Predictive intelligence** No predictive intelligence capability	**Predictive intelligence** Predictive intelligence capability to inform and help plan capacity	**Predictive intelligence** Predictive intelligence capability to inform and help plan capacity
	**Fee** £250	**Fee** £250	**Fee** £1500
**Prob. of purchase**	**9%**	**89%**	**80.5%**
**Prob. to decline purchase**	**91%**	**11%**	**19.5%**

### Basic cost-benefit evaluation of the dashboard online tool

We performed a basic cost-benefit simulation exercise to evaluate the profitability of a dashboard tool venture, using the three dashboards from Table 8, i.e. the baseline, a fully-featured low price and a fully-featured high price alternative. We assumed the dashboard is offered to 100, 500 and 1000 potential interested clients (a range chosen to highlight the possible profitability of the dashboard in a hypothetical market). The product up-take was calculated from Table 8 to determine purchasing behaviour (research question 3). For the calculation of costs, we only took into account the initial development cost (i.e. the value of the overall grant of £412,242, which encompassed various small studies culminating in development of the toolkit) and assumed a varying annual maintenance cost depending on the number of clients (i.e. £50 K for 100 potential clients, £100 K for 500 potential clients, £150 K for 1000 potential clients). Finally, we assumed a product life of 5 years and an interest rate of 3%.

From Table 9 it is apparent that the baseline dashboard is not a viable option for any of these numbers of potential clients, largely due to its very low chances of uptake (i.e. even for 1000 potential clients, only 90 purchases are predicted). Moving on to the full-featured option with the low pricing strategy, the product becomes profitable over a 5 year period only for 1000 potential clients (i.e. 890 purchases per year). Finally, for the full-featured option in the high pricing strategy the product becomes highly profitable for 500 potential clients (i.e. 403 purchases per year) and more so for 1000 potential clients (i.e. 805 purchases per year).

**Table 9:** Basic cost-benefit evaluation of a dashboard tool. Data generated using JMP software Version 13.0

Revenues	Baseline	Fully-featured low price	Fully-featured high price
Probability of purchase (out of potential clients)	9.0%	89.0%	80.5%
Product price	£250	£250	£1500
Annual revenue			
for 100 potential clients	£2250	£22,250	£120,750
for 500 potential clients	£11,250	£111,250	£603,750
for 1000 potential clients	£22,500	£222,500	£1,207,500
Present value of total revenue (over a period of 5 years with an interest rate of 3%)			
for 100 potential clients	**£10,304**	**£101,898**	**£553,000**
for 500 potential clients	**£51,522**	**£509,492**	**£2,764,998**
for 1000 potential clients	**£103,043**	**£1,018,985**	**£5,529,996**
**Costs**			
Development Cost	£412,242.00		
	For 100 potential clients	For 500 potential clients	For 1000 potential clients
Annual maintenance cost	£50,000	£100,000	£150,000
Present value of total costs (over a period of 5 years with an interest rate of 3%)	**£641,227**	**£870,213**	**£1,099,198**

Notes

- A product life of 5 years is assumed

- Interest rate of 3%

### Synthesising the DCE results with those from previous and subsequent stages of the PRESENT project

In the final part of the PRESENT study we wished to consider how people actually used our working prototype, refined following our DCE. We observed 13 NHS managers at their workplaces in three diverse parts of England as they ‘walked through’ the dashboard to answer a relevant hypothetical question about cancer care. Each walkthrough and an associated interview lasted on average 2 h and was videoed. Walkthroughs were structured according to published guidance [15, 19] and designed to elicit information on our system’s heuristics, usefulness in finding the answer to the hypothetical question (each participant devised their own question), and implementation barriers and enablers. This approach, which was different to the unstructured cognitive interviews used in our questionnaire development, meant we evaluated the quality of the user interface and system behaviour, not the actions of participants.

At the end of this stage we used a triangulation matrix to compare findings across the different parts of the study as a synthesis of study findings on dashboard attributes. Methods were the columns and attributes the rows. We used this matrix to examine convergences and divergences, which included a return to the raw data, to gain a deeper understanding of the requirements of our potential end users and the different types of data afforded by the different methods used.

Searches and filters were important across our data but the suggested form they should take differed according to the approach we used to explore them, as did the colours to use in the charts (red-amber-green or a more neutral scheme) and the use of maps to indicate patterns in the data. Our final synthesis summary can be seen in Table 10.

**Table 10:** Summary of our final synthesis of the different sources of data in the PRESENT study, including the DCE

Design features	Subsets	Source of data				Consensus across data sources
		Systematic review	Concept mapping (Pts = 29, HCPs = 6)	Walkthrough (HCPs=13)	DCE (n=32, mixed) NB DCE ranked features rather than determining them	
Uses	Patients	Simple to follow, clear layout which anticipates user’s workflow and needs (goal directed); time-saving	Support, planning, comparisons, choice	Identify areas of need Plan improvements Seek funding Show successes Time-saving	Option to tailor themes of relatively low priority	Broad agreement across sources
	HCPs		Identify areas of need Plan improvements			
Timely data		N/A	Important	Important	n/a	Agreement from two sources
Functions	Navigation	Clear and simple Labelling of buttons Shortcuts Avoid scrolling	Simple Scrolling OK	Clear and simple Help prompts / pop-up illustrations on use Shortcuts Scrolling OK	n/a	Some agreement, some disagreement
	Search and Filtering	Ability to personalise and tailor depending on needs and preferences Multiple comparisons	Keyword search Highly customisable filtering Compare hospitals /staff Dislike dropdown lists	Keyword search Multiple filtering options (e.g. demographic) Need to see non-responders Compare hospitals and wards	Dropdown menus slightly preferred over keyword search, both important Multiple filtering options important	Some agreement, some disagreement
	Export	The option to print/download is valued and desirable to both Pts and HCPs	Print options	Need export / report function	n/a	Agreement from three sources
Presentation	Colours	RAG liked (bright, distinct and highly contrasting colours)	RAG colours disliked	RAG liked	n/a	Disagreement between sources
	Drill-down	Important	Important	Important	n/a	Agreement from three sources
	Maps	n/a	Not liked	n/a	Maps relatively preferred	Disagreement between sources
	Infographics and data	Line and bar charts clear and appropriate for providers ‘at a glance’ overviews	Pie and bar charts % and raw numbers Summary overview of themes	Pie or bar chart %, raw numbers and denominators Summary overview	n/a (function to change chart type relatively unimportant)	Broad agreement across sources
	Language and feel	Simple language without abbreviations and jargon Images informative, not solely decorative	n/a	Match to user group – photos, words Minimal but informative text Help/prompts needed on dashboard (low priority)	Relatively low priority	Broad agreement across sources
Data integrity	Data validity & labels	Elements all well explained	n/a	Validity shown Elements all well explained	n/a	Agreement from two sources
	Security Access	Easy access & simple URL Stand-alone systems/ separate login less likely to be used But security must be prioritised	Registration can put people off accessing	Open to all. Passwords will deter	n/a	Agreement from three sources
Other	Community function	Forum, chat room and similar community features	Forum, chat room and similar community features	n/a	n/a	Agreement from two sources
	Other information	Signposting to other sources of information and support Dashboards that offer solutions to be adopted	n/a	Links to other guidance good, if well chosen	n/a	Agreement from two sources
	Adding more data	n/a	User input possible	n/a	Upload own data (but predictive capability relatively unimportant)	Agreement from two sources

## Discussion

In this paper we have presented the development of an online questionnaire DCE to elicit individual preferences for features for an online dashboard, and explored results. The choice of an online questionnaire for our experiment was appropriate given that a technology was being assessed. We were not interested in the exact content of the dashboard, which had been determined from previous stages of the study [1], but on the availability of features and customisation options and how they affect individuals’ purchasing behaviours. In other words, the purchasing behaviour elicited in this context assumes that individuals are already aware of the importance of the information offered by the online dashboard and as such we assess how the various features of such a dashboard influence the probability of purchasing the product. In this way we can determine which features are the most important and triangulate these with the results from other forms of assessment. At the same time, we allowed individuals to opt-out of purchasing, which affords the opportunity to elicit a demand function for the product. Individuals could opt-out because they disliked one or more features of the product.

The DCE is a useful tool in the development and evaluation of this type of intervention as it renders feasible an assessment of the intervention’s dimensions prior to its release in the market. Findings suggest that certain features are highly desirable, namely the search function, filtering, and upload own data being the top choices. Further, a range of WTP values is observed, mostly towards the upper end of the distribution that was specified in the price attribute, again suggesting that certain features were highly valued and would contribute significant added value in a potential dashboard. Finally, in terms of the market demand for three representative dashboards we found purchasing behaviour to be very much dependent on the dashboard features, going from a 10 to 90% probability to purchase when we moved from a baseline dashboard to a fully-featured one.

In the preliminary and developmental parts of the study (stages 1 and 2) we effectively asked stakeholders for their considered impressions of what they believe they are doing in preattentive processing (i.e. subconscious cognitive processes) and we explored this in our scoping review. It could be argued that our DCE work attempted to get at preattentive processing more objectively. Therefore the information obtained from this DCE was particularly helpful for refining the prototype dashboard developed for the PRESENT study. Since all features were desired to at least some degree, and we could not list them in order of importance from other stages of the study, the DCE was critical in determining which features were least desired. This meant we were able to make decisions on which features to prioritise in further development. This was especially useful when programming constraints meant we could not have particular combinations of features in our final prototype or when there was lack of clarity from our other data. The DCE also gave the team confidence in the desirability of the dashboard. Results were also used, with data from other parts of the study, to develop a checklist of desirable healthcare dashboard features. The holistic approach that we used in PRESENT to co-create our dashboard, incorporating human, organisational, economic and technology dimensions, is increasingly recognised as necessary. This is shown for example by the development of holistic, evidence based frameworks such as that of the Centre for eHealth Research and Disease Management (CeHRes) [20] for end-to-end design, development and implementation of health informatics technologies.

There is nothing particular about a healthcare dashboard that would make it a specific candidate for the use of DCE in the way we have described per se, but healthcare dashboards have types of data, uses and historical contexts that make their attributes different to those of other dashboards. Thus our use of DCE can be generalised beyond healthcare but the data from our study are only potentially generalisable to other healthcare management dashboards.

While the DCE proved useful in helping the developers decide on the core features for the PRESENT dashboard, extrapolating actual market demand from the experimental findings should be treated with caution for various reasons. First, while a response rate cannot be calculated in online questionnaires, we have a moderate retention rate of about 20%. It is unknown whether those who logged into the questionnaire but did not complete did not do so because they were not interested in the product, not interested in participating or not able to devote the required time, or for some other reason. At the same time, our sample is small, potentially affecting generalisability of preferences and findings. Second, we included snowball sampling with people who had taken part in the study, who may have been biased in favour of the dashboard; however, the nature of the DCE and the large number of invitations sent to other relevant people through professional organisations mean this bias is diluted. Nonetheless, it is not clear what the uptake would be among the overall stakeholder population. Third, while we made every effort to appear realistic in the way we presented the dashboard and its features to participants, the DCE itself and the purchasing behaviour is of a hypothetical nature, where individuals express intentions. There is some literature to suggest that discrepancies between stated and actual behaviours are not uncommon [21] and DCE results are to be verified and further replicated before generalisations can be made. Fourth, in general individuals are assumed throughout to be perfect information processors, although this is not always the case [22, 23]. Moreover, the problems of potential inconsistency of the respondents’ answers to the hypothetical situations increase with the size of the experiment and the difficulty of the tasks [24, 25]. Often individuals find answering the necessary questions increasingly difficult, while fatigue also sets in [26]. These problems could inhibit individuals from using compensatory behaviour (trading off one attribute for another), and instead lead them to either answer at random or use other types of non-compensatory techniques, which could result in lack of robustness in the results [22, 27].

Overall, with these concerns in mind, and with a few further assumptions, a basic evaluation exercise suggests that the development of the online dashboard and its roll-out in the market would result in a positive net benefit and suggests which features are the most important to develop in such a dashboard. We should highlight that this cost-benefit analysis offers a lower bound estimate of the net benefit as it does not acknowledge or incorporate any of the non-monetary benefits that would result from the use of the online dashboard and from which the main benefits are expected to arise (i.e. improved healthcare services, improved health outcomes, enhanced data availability and research etc.). However, given that such evaluation is outside the scope of this project, this would have to be explored in future research.

## Conclusion

DCEs can be successfully used to inform development of an online dashboard by determining preferences for particular features and customisation options and how this affects individuals’ purchasing behaviours. This provides an objective way of determining preferred technology features that is based on access to subconscious cognitive processes. The approach should be transferable to the development of other technologies.

## Supplementary information

**Additional file 1.** The final list of attributes and levels selected for the DCE.

## Data Availability

These may be obtained from the corresponding author on request.

## Abbreviations

CPES: National Cancer Patient Experience Survey
CeHRes: Centre for eHealth Research and Disease Management
CL: Conditional logit
DCE: Discrete Choice Experiment
MRS: Marginal rates of substitution
NHS: National Health Service (UK)
NL: Nested logit model
NRES: National Research Ethics Committee
p.p.: Percentage points
UK: United Kingdom
WTP: Willingness-to-pay
