# Supplementary material for: A proof-of-concept framework for the preference elicitation and evaluation of health informatics technologies: the online PRESENT patient experience dashboard as a case example
Source: BMC Med Inform Decis Mak. 2020 May 24;20:95. doi: 10.1186/s12911-020-1098-z (PMC7245892; doi:10.1186/s12911-020-1098-z)
Supplement: Supplementary file 1 — Additional file 1. The final list of attributes and levels selected for the DCE. [file 12911_2020_1098_MOESM1_ESM.docx]

| **Supplementary information: The final list of attributes and levels selected for the DCE** |
| --- |
| **Search**   1. **Option to search for data on a specific hospital OTHER THAN YOUR OWN:**  - Yes: choose a hospital from a drop down list - Yes: keyword search [type in part of the word and suggestions appear] - Yes: map and postcode [choose from a map or type in postcode to see the list of hospitals in the area] - No: Only regional data visible   **Presentation**   1. **Graphs:**  - Fixed graphs or pie charts - User can choose and change graphs  1. **Data resolution:**  - Show graphs and pie charts only if more than 6 responses, but show all comments - Show graphs and pie charts as well as comments even if there is only 1 comment for the chosen topic   **Display**   1. **Language:**  - Technical, with a dictionary of terms - Lay, that is with no jargon  1. **Indicators displayed:**  - 6 fixed indicators of patient experience shown at the same time - User can choose up to 6, out of 12 indicators of patient experience     **Data breakdown**   1. **Filter**  - Filter data by gender AND age AND ethnicity AND condition/illness - Filter only by condition or illness (e.g. type of cancer)  1. **Staff role**  - Filter comments by staff role - Do not filter by staff role  1. **Upload your own data:**  - Yes - No   **Other**   1. **Predictive intelligence (see how lack of given resources may influence particular areas of healthcare)**  - Predictive intelligence capability to inform and help plan capacity - No predictive intelligence capability   **Fee**   1. **Annual team membership cost**  - £250 - £500 - £1,000 - £1,500 |
